# Transplantation of bone marrow-derived mononuclear cells can improve the survival rate and suppress the inflammatory response in a rat crush injury model

**DOI:** 10.1186/cc13255

**Published:** 2014-03-17

**Authors:** J Nakagawa, N Matsumoto, K Yamakawa, T Yamada, H Matsumoto, T Muroya, H Ogura, T Shimazu

**Affiliations:** 1 Osaka University Graduate School of Medicine, Suita-shi, Osaka, Japan

## Introduction

Crush syndrome is often encountered in natural disasters. It is a critical condition leading to multiple organ failure. However, the mechanisms by which the local traumatic injuries affect distant organs remain unknown. We paid attention to bone marrow-derived mononuclear cells (BMMNCs) as therapeutic strategy against crush injury. Transplantation of BMMNCs can elicit protection and regeneration against damaged organs through their paracrine properties and its clinical application has been realized to treat ischemia reperfusion-related various diseases. We have previously reported that multiple organ damage based on systemic inflammatory response is induced in crush injury and the pathogenesis is largely dependent on massive ischemia-reperfusion. We investigated whether BMMNCs could suppress systemic inflammation and improve mortality in a rat model of crush injury.

## Methods

To develop crush syndrome, both hind limbs of rats were compressed for 6 hours under weights (3.0 kg each). Rats in the treated group were intravenously administrated BMMNCs (1 × 10^7 ^cells in 1 ml phosphate-buffered saline (PBS)) immediately after weight removal (BMMNC group) and PBS alone was administered in the control group (CR group). The sham group underwent the same procedure without compression of hind limbs (SH group). The rats were observed over 7 days after the injury to evaluate survival. To estimate antiinflammatory effects of BMMNCs, sera were collected 3 hours, 6 hours and 24 hours after the injury. The levels of interleukin 6 (IL-6) and tumor necrosis factor alpha (TNFa) were measured by ELISA and statistically analyzed.

## Results

The survival rate at day 7 in the BMMNC group was 80.0%, and that in the CR group was 47.6%, revealing that the 7-day survival was significantly improved by the BMMNC injection (*P *< 0.05). Crush injury-induced upregulation of serum IL-6 was significantly reduced by the BMMNC treatment at all time points (*P *< 0.05). The level of TNFa decreased significantly in the BMMNC group compared with that in the CR group 24 hours after the compression release (*P *< 0.05). These findings suggest that transplantation of BMMNCs has an ability to evade the devastating condition following crush injury by suppressing systemic inflammation.

## Conclusion

The administration of BMMNCs reduced production of inflammatory cytokines and improved survival rate in a rat model of crush injury. Cell therapy using BMMNCs might become a novel therapy against crush injury.

